# A randomized home-based childhood obesity prevention pilot intervention has favourable effects on parental body composition: preliminary evidence from the Guelph Family Health Study

**DOI:** 10.1186/s40608-019-0231-y

**Published:** 2019-03-04

**Authors:** Owen Krystia, Tory Ambrose, Gerarda Darlington, David W. L. Ma, Andrea C. Buchholz, Jess Haines, on behalf of the Guelph Family Health Study

**Affiliations:** 1 Department of Family Relations and Applied Nutrition, University of Guelph, Guelph, ON N1G 2W1 Canada; 2 Department of Mathematics and Statistics, University of Guelph, Guelph, ON N1G 2W1 Canada; 3 Department of Human Health and Nutritional Sciences, University of Guelph, Guelph, ON N1G 2W1 Canada

## Abstract

**Background:**

Home-based lifestyle behaviour interventions show promise for treating and preventing childhood obesity. According to family theories, engaging the entire family unit, including parents, to change their family behaviour and dynamics may be necessary to prevent the development of childhood obesity. However, little is known about how these interventions, which may change the family dynamics and weight-related behaviours of parents, affect weight outcomes in parents. Our objective was to examine the effect of a pilot home-based childhood obesity prevention intervention on measures of anthropometrics and body composition in Canadian parents.

**Methods:**

Forty-four families with children aged 1.5–5 years were randomized to one of three groups: 4 home visits with a health educator, emails, and mailed incentives (4 HV); 2 home visits, emails, and mailed incentives (2 HV); or general health emails (control). Both the 2 HV and 4 HV intervention were conducted over a period of 6 months. Body composition and anthropometric outcomes were measured at baseline and at 6 months and 18 months from baseline.

**Results:**

In parents with baseline body mass index (BMI) ≥ 25 kg/m^2^, the 2 HV group had significantly lower body mass and waist circumference at 6-month (CI = -5.85,-0.14 kg;-5.82,-0.30 respectively) and 18-month follow-up (CI = -7.57,-1.21 kg;-9.30,-2.50 cm respectively) when compared to control, and significantly lower BMI at 18-month follow-up when compared to control (CI = -2.59,-0.29 kg/m^2^). In parents with baseline BMI < 25 kg/m^2^, the 4 HV group had significantly lower percentage fat mass (CI = -3.94,-0.12%), while the 2 HV group had significantly lower body mass (CI = -2.56,-0.42 kg) and BMI (CI = -0.77,-0.08 kg/m^2^) at 6-month follow-up, both compared to control; these effects were not maintained at 18-month follow-up.

**Conclusions:**

This study provides support that a home-based childhood obesity prevention intervention may improve weight outcomes among parents. Future research should explore how home-based interventions influence family behaviour and dynamics to impact weight outcomes in children and their parents.

**Trial registration:**

Prospectively registered August 2014**,** clinical trial identifier NCT02223234.

## Background

In Canada, the high prevalence of obesity among children and adults is an important public health issue [1]. Previously, a number of modifiable behaviours have been found to influence the development of obesity in children, such as screen time, sleep time, and dietary intake [2–4]. However, research suggests that children are not achieving recommendations for these weight-related behaviours [5–7]. Early intervention in childhood when weight-related behaviours are formed may prove most successful in preventing obesity [8, 9]. To change weight-related behaviours in children, we must target and engage the caregivers of children, i.e., their parents [10]. This is complicated by the fact that parents may themselves be overweight or obese, suggesting that family environments may be obesogenic in nature.

Interventions which incorporate parents into childhood obesity preventions are termed family-based interventions [11, 12]. Family-based childhood obesity interventions may be effective at preventing and treating childhood obesity [13]. Based on the results of obesity prevention trials conducted in Australia and America, a 2016 Institute of Medicine report identified home-based obesity interventions, a type of family-based intervention conducted in the home setting, as one of the most promising strategies in the prevention of childhood obesity [14–17]. Behavioural interventions which are conducted within the home setting can be particularly tailored to a family’s unique dynamics and living situation. These interventions may encourage changes in parental health-related behaviours and parenting practices, which may lead to improved weight outcomes in children [10–12].

Multiple family theories inform family-based obesity interventions, many of which have overlapping concepts [11]. Many such theories posit that families are systems with interdependence of units [11]. For example, the Family Systems Theory posits that a change in one element of the family will have an impact on another [18]. Thus, if a program intervenes to change parenting practices and behaviours which are protective against the development of childhood obesity, such as limiting children’s screen time, it is possible that parents may also adopt these behaviours, thereby affecting parental weight outcomes. By improving weight outcomes in parents through a childhood obesity prevention intervention, a family-based intervention would benefit both children and their parents. Obesity is associated with many negative health outcomes, making improvements in parent weight beneficial. However, limited research has investigated how childhood obesity prevention interventions may impact parents, including their measures of anthropometrics and body composition.

To our knowledge, only one study has investigated parental body composition changes in a family-based childhood obesity prevention intervention. This study conducted a community-based intervention to improve physical activity among Australian fathers who were overweight/obese and their school-aged children in order to prevent the development of obesity in these children [19]. Both body mass index (BMI) and waist circumference (WC) decreased among fathers in the intervention group. No study has examined how a home-based intervention that targets childhood obesity prevention may impact both fathers’ and mothers’ weight outcomes. Additionally, investigating the influence of interventions on the amount and distribution of body fat, rather than proxy measures of overall body composition, such as BMI, would provide a more differentiated view on the effects of these interventions on parents’ adiposity.

The Guelph Family Health Study (GFHS) pilot tested a home-based childhood obesity intervention prevention informed in part by the Family Systems Theory [20]. The GFHS intervention is based on the Healthy Habits, Happy Homes Intervention, a home-based obesity prevention intervention which improved weight-related behaviours among young children of American families [15]. Preliminary evidence from the GFHS pilot found that the home-based obesity intervention was feasible in the Canadian context and may lead to improved diet and weight outcomes of children [20, 21]. As both the Healthy Habits, Happy Homes Intervention and the GFHS lead to successful adoption of protective weight-related behaviours in children, this suggests that the broader family system, including parents of these children, may be adopting healthful behaviours [11]. However, neither study has of yet investigated the impact of the home-based intervention on parent outcomes.

Our objective was to examine the effect of a 6-month home-based, childhood obesity prevention pilot intervention on parents’ anthropometrics and body composition. The primary outcome of the GFHS pilot study was prevention of childhood obesity by intervening on weight-related behaviours using a family based approach [20]. The current study is formative and investigates if improvements in parental adiposity is a beneficial secondary outcome of a childhood obesity prevention intervention. In turn, results of this study may illustrate the relevance of Family Systems Theory for obesity prevention interventions [11, 18].

## Methods

### Study design

The Guelph Family Health Study (GFHS) pilot is a three-arm external pilot randomized control trial among 44 families with at least one preschool-aged child (*n* = 79 parents and 53 children), residing in Wellington County, Ontario, Canada (clinical trial identifier: NCT02223234). The design, feasibility, and preliminary impact related to child outcomes for the GFHS have been previously reported [20]. During recruitment, families were excluded from enrolment if they planned to move within one year of the start of the study, had children outside of the target age range (1.5–5 years), or were non-English speaking. All study procedures took place at the University of Guelph from 2014 to 2016 after the parents provided written, informed consent. The study was approved by the University of Guelph Research Ethics Board (REB14AP008/REB14AP009).

### Study groups

**Fig. 1:**
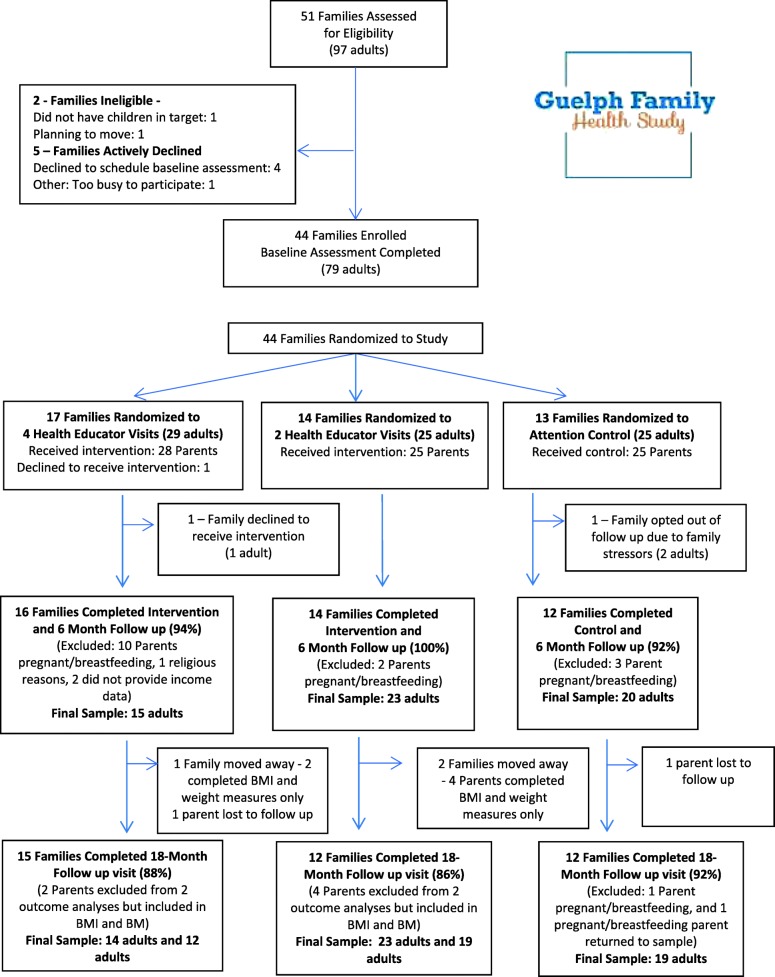
Study design and participant flow of the Guelph Family Health Study Pilot and analytic sample

After a baseline assessment, the study coordinator used a pseudo-random number generator to randomly assign each family into one of three groups: four home visits with a health educator, tailored emails, and mailed incentives (4 HV; *n* = 17 families); two home visits with a health educator, tailored emails, and mailed incentives (2 HV; *n* = 14 families); or general health advice through emails (control; *n* = 13 families) (Fig. 1).

#### GGuelph Family Health Study intervention: 4 HV and 2 HV

The GFHS intervention is theoretically informed by both the Family Systems Theory [18] and the Self-Determination Theory [22]. Families randomized to the two intervention arms received either 4 or 2 home visits with a health educator (HE) trained in motivational interviewing [23], in addition to weekly e-mails and monthly mailed items to support behavioural changes (e.g., a children’s book to support improved sleep-time goals). Previously, motivational interviewing has been found to support the autonomy required for behaviour change as posited by Self-Determination Theory [24]. During home visits, HEs provided support around adopting and maintaining healthful family routines (e.g., limiting children’s sugar-sweetened beverage consumption, engaging in family physical activity, establishing sleep routines to increase child sleep duration, limiting the children’s sedentary time, and having more family meals). During follow-up visits, HEs reviewed progress and helped families set new behaviour change goals, if applicable. Initial HE visits lasted 1 h; follow-up visits lasted between 30 and 60 min. The intervention was delivered over a period of 6 months.

#### Minimal-attention control

Families randomized to the control group received monthly emails containing publicly-available information on general health (e.g., current Canadian physical activity guidelines).

### Measures

All measures were recorded at baseline, at 6 months from baseline (at the completion of the intervention) and 18 months from baseline (1-year post intervention). To minimize risk of bias, staff who conducted measurements were not involved in the delivery of the intervention. Measurements were collected at the Body Composition Laboratory at the University of Guelph.

#### Anthropometrics

Parent height was measured, in duplicate, using a wall-mounted stadiometer (Medical Scales and Measuring Devices; Seca Corp., Ontario, California, USA). Body mass (BM) was measured using the BOD POD™ digital scale (Cosmed Inc., Concord, California, USA). BMI was calculated as weight (kg)/height^2^ (m^2^). Waist circumference (WC) was measured, in duplicate, at the iliac crest, at mid-respiration using a non-elastic measuring tape (Gulick II, Country Technology Inc., Gay Mills, Wisconsin, USA), per the protocol from Statistics Canada and the Canadian Health Measures Survey [25].

#### Body composition

Body composition was determined using air displacement plethysmography (ADP) with a BOD POD following the manufacturer-recommended protocol. ADP measures total body volume, from which whole-body density and, subsequently, percentage fat mass (%FM) are calculated [26].

The intra-individual variation of repeated %FM measures was 2.1%, within the acceptable variation range of 1.7 to 4.5% [27]. The inter-rater coefficient of variation was 2.6%, falling below the reference value of 2.7% [27].

### Analytic sample and statistical analyses

Data from the GFHS were excluded from the analytic sample if participants were unable to provide anthropometric or body composition measures (*n* = 15 pregnant/breastfeeding at both 6- month follow-up and 18-month follow-up, n = 1 religious exclusion), failed to provide income data (*n* = 2), or if participants were lost to follow-up (*n* = 3 at 6 months and 2 at 18 months). The final sample consisted of 60 parents at baseline, 58 parents at 6 months, and 56 parents at 18 months (6 months: 4 HV *n* = 15, 2 HV *n* = 23, control *n* = 20; 18 months: 4 HV *n* = 14, 2 HV *n* = 23, control *n* = 19) (see Fig. 1 for participant flow).

Analyses were conducted using SAS® University Edition for OS X (SAS Institute Inc., 2015). To assess the effect of the intervention on parental body composition outcomes, a linear regression model was created to examine follow up outcomes in BM, BMI, WC, and %FM, comparing the intervention groups to control, at 6 M and 18 M while accounting for baseline anthropometrics and body composition values. Generalized estimating equations were used to account for dependent observations within families [28]. Differences by baseline BMI status (≥ 25 vs. < 25 kg/m^2^) were observed when the groups were analyzed separately, so we present stratified results only. There was no evidence of moderation by parent sex, so the analysis was not stratified by sex (data not shown). Parent age, sex, household income, and baseline anthropometric and body composition measures were entered as covariates in all models. Significance was identified at *p* < 0.05. The GFHS pilot was not designed as a fully powered study.

## Results

Approximately 86% of parent participants identified as White. The average age of participants was 35 and the sample included 33 fathers (55%) (Table 1). On average, participants were classified as overweight (mean BMI = 28.3 ± 6.8 kg/m^2^), with no significant baseline differences between stratified intervention groups with respect to anthropometrics or body composition (*p* > 0.05).

**Table 1:** Baseline characteristics of parent participants from the Guelph Family Health Study pilot analytic sample and by intervention assignment

	Overall	Control	Intervention	
	(*n* = 60)	(*n* = 21)	2 HV (n = 23)	4 HV (*n* = 16)
Relation to child, n (%)				
Mother	27 (45.0)	10 (47.6)	12 (52.2)	5 (31.3)
Father	33 (55.0)	11 (52.4)	11 (47.8)	11 (68.8)
Baseline age, mean (SD)	35.3	34.5 (2.8)	35.7 (3.2)	35.7 (4.4)
Total annual household income^a,b^, n (%)				
Less than $60,000	12 (28.6)	4 (33.3)	4 (28.6)	4 (25.0)
$60,000 to $99,999	13 (31.0)	3 (35.0)	5 (35.7)	5 (31.3)
$100,000 or more	16 (38.1)	5 (41.7)	5 (35.7)	6 (37.5)
Body composition, mean (SD)				
Body Mass, kg	85.1 (24.5)	80.3 (16.9)	83.9 (23.4)	95 (33.7)
BMI ≥ 25 kg/m^2^		88.58 (16.37)	98.94 (21.59)	109.53 (32.71)
BMI < 25 kg/m^2^		66.87 (4.75)	67.39 (10.98)	66.51 (8.18)
BMI, kg/m^2^	28.4 (6.9)	27.7 (5.1)	27.8 (6.8)	30.6 (8.64)
BMI ≥ 25 kg/m^2^		30.65 (5.01)	32.58 (5.95)	34.17 (8.02)
BMI < 25 kg/m^2^		22.91 (1.12)	22.52 (1.83)	22.07 (1.19)
WC, cm	97.5 (17.5)	94.3 (12.8)	96.1 (16.8)	104.7 (22.4)
BMI ≥ 25 kg/m^2^		99.77 (12.67)	108.48 (14.59)	113.02 (23.63)
BMI < 25 kg/m^2^		85.36 (6.65)	83.77 (6.50)	87.42 (7.06)
%FM	30.8 (11.0)	29.5 (10.4)	31.3 (11.3)	32.5 (12.0)
BMI ≥ 25 kg/m^2^		21.79 (11.18)	38.56 (7.64)	35.81 (10.37)
BMI < 25 kg/m^2^		25.64 (8.11)	23.40 (9.11)	22.54 (11.06)

^a^Categories have been condensed to protect participant and family ID

^b^Family level variable

At both 6-months and 18-months from baseline, parents with a BMI ≥ 25 kg/m^2^ at baseline were observed to have significant reductions in both body mass and WC in the 2 HV group, as compared to control (Table 2). At 6-months, body mass was on average 2.99 kg lower (CI = − 5.85, − 0.14 kg), and at 18-months, was 4.39 kg lower (CI = − 7.57, − 1.21 kg), when compared to the control group. At 6-months, WC was on average 3.06 cm lower (CI = − 5.82, − 0.30 cm), and at 18-months, was 5.90 cm lower (CI = − 9.30, − 2.50 cm), when compared to control. In the 2 HV group, BMI did not significantly change from baseline to 6-months as compared to control, but at 18-months BMI was 1.44 kg/m^2^ lower compared to control in this same group (CI = − 2.59, − 0.29 kg/m^2^). In the 4 HV intervention group, parents with a BMI ≥ 25 kg/m^2^ at baseline observed no significant reductions or increases in any anthropometric or body composition variables at either time point.

**Table 2:** Linear regression model results comparing intervention groups to control with respect to body composition outcomes among parents participating in the Guelph Family Health Study at 6-months and 18-months post-intervention controlling for baseline body composition values and stratified by BMI status

Outcome		0 to 6 months (completion of intervention)		0 to 18 months (1-year post intervention)	
		$\widehat{B}$ ^a^	*p*-value (95% CI)	$\widehat{B}$ ^a^	*p*-value (95% CI)
Analysis of treatments (4 HV *n* = 9 parents at 6 M and *n* = 8 parents at 18 M; 2 HV *n* = 12 parents at 6 M and 18 M) compared to control group (*n* = 13 parents at 6 M and *n* = 12 at 18 M) for participants with baseline BMI ≥25.0 kg/m^2^					
Body mass, kg	2 HV vs. control	**−2.99**	**0.04 (− 5.85, − 0.14)**	**−4.39**	**< 0.01 (− 7.57, − 1.21)**
	4 HV vs. control	− 0.02	0.90 (− 2.79,2.45)	−0.27	0.89 (− 4.04, 3.50)
BMI, kg/m^2^	2 HV vs. control	−1.02	0.06 (− 2.06, 0.03)	**− 1.44**	**0.01 (− 2.59, − 0.29)**
	4 HV vs. control	0.17	0.72 (− 0.76, 1.10)	− 0.02	0.98 (− 1.22, 1.19)
WC, cm	2 HV vs. control	**−3.06**	**0.03 (−5.82, − 0.30)**	**−5.90**	**< 0.01 (− 9.30, − 2.50)**
	4 HV vs. control	0.18	0.89 (− 2.20, 2.55)	− 1.58	0.28 (− 4.43, 1.27)
FM, %	2 HV vs. control	− 2.51	0.05 (− 5.06, 0.05)	− 2.72	0.17 (−6.58, 1.15)
	4 HV vs. control	− 0.42	0.73 (− 2.82, 1.98)	− 1.40	0.43 (− 4.85, 2.05)
Analysis of treatments (4 HV *n* = 5 parents; 2 HV *n* = 11 parents at both 6 M and 18 M) compared to control group (*n* = 8 parents at both 6 M and 18 M) for participants with baseline BMI < 25 kg/m^2^					
Body mass, kg	2 HV vs. control	**−1.49**	**< 0.01 (−2.56, − 0.42)**	0.50	0.74 (− 2.46, 3.46)
	4 HV vs. control	−0.95	0.24 (−2.54, 0.64)	−0.16	0.88 (− 2.16, 1.84)
BMI, kg/m^2^	2 HV vs. control	**−0.42**	**0.02 (−0.77, − 0.08)**	0.10	0.85 (− 0.92, 1.11)
	4 HV vs. control	−0.34	0.21 (− 0.87, 0.19)	−0.16	0.69 (− 0.95, 0.63)
WC, cm	2 HV vs. control	−0.65	0.68 (−3.70, 2.41)	−1.88	0.33 (− 5.68, 1.91)
	4 HV vs. control	−0.40	0.85 (−4.57, 3.77)	−1.13	0.58 (−5.16, 2.90)
FM, %	2 HV vs. control	0.99	0.34 (−1.04, 3.01)	0.62	0.76 (−3.27, 4.50)
	4 HV vs. control	**−2.03**	**0.04 (−3.94, −0.12)**	0.24	0.89 (−3.19, 3.66)

^a^Models adjusted for baseline age, sex, family household income, and baseline body composition measurement

Sample size for each outcome may vary slightly due to missing data

Bolded values *p* < 0.05

Among parents with BMI < 25 kg/m^2^ at baseline, no significant increases in anthropometrics or body composition were observed as compared to control in either the 2 HV or 4 HV intervention group at either time point. As an effect of the intervention, significant reductions in body mass and BMI were observed at 6-months in the 2 HV group when compared to control ($\widehat{B\ }$= − 1.49 kg, CI = − 2.56, − 0.42 kg; $\widehat{B\ }$= − 0.42 kg/m^2^, CI = − 0.77, − 0.08 kg/m^2^; Table 2). However, these results were not sustained at 18-months from baseline. In the 4 HV group, at 6-months from baseline, %FM was on average 2.03% less (CI = − 3.94, − 012%) as compared to control. This decrease was not sustained at 18-months from the baseline measurement.

## Discussion

This research provides preliminary evidence that sustained improvements in parental adiposity may be a beneficial secondary outcome of a home-based childhood obesity prevention intervention. This is a unique contribution to the literature as this is the first study to examine parental body composition as a secondary outcome of a family-based childhood obesity-prevention intervention situated in the home setting rather than in the community. In addition, these findings provide preliminary evidence that childhood obesity preventions may lead to changes in family dynamics and parental behaviours, which in turn improve parental weight outcomes.

It is difficult to contextualize our results given the dearth of research on parental body composition-related outcomes of family-based obesity prevention interventions. In a 2014 Australian family-based obesity prevention-intervention conducted in a community setting, fathers who were overweight/obese were observed to have significant reductions in BMI (1.0 kg/m^2^) and WC (~ 4.0 cm) compared to control at 14-weeks post-intervention [19]. These reductions represent short-term changes which are greater in magnitude than the 6-month results of our intervention but are of similar magnitude to the 18-month results in our overweight/obese participants. The reductions in parent anthropometrics in the Australian study may relate to the intervention’s focus on increasing parent physical activity (PA) and improving diet quality [19]. The GFHS intervention focuses on establishing and maintaining healthful behaviours, tailored to the families’ interests, which may or may not include PA [20]. These behaviour changes may take longer to reveal significant effects on parent weight-related outcomes and may help to explain why BMI reductions were experienced in the 2 HV group at 18-months compared to baseline, but not at 6-months compared to baseline. Alternatively, parents may have changed their behaviours only after the 6-month intervention.

Literature reporting parental body composition changes in family-based child obesity *treatment* interventions, compared to *prevention* interventions, is more common. These interventions, which target children who are overweight/obese, have reported beneficial changes in parent body composition [29–31]. In the present study, we report BMI reductions of 1.44 kg/m^2^ at 18 M in the 2 HV group, which is consistent with decreases in parental BMI reported in previous childhood obesity treatment interventions, which range from 0.5 kg/m^2^ to 2.0 kg/m^2^ at similar intervention time points [29–31]. The GFHS intervention expands on these previous findings by reporting multiple measures of anthropometrics and body composition.

Our results suggest that similar to adult-focused treatment interventions, family-based childhood obesity prevention interventions may result in beneficial outcomes with regards to parent body composition. Previous adult-focused obesity interventions have also demonstrated changes in body composition similar to the those observed in our study [32, 33]. A meta-analysis of adult behavioural obesity interventions in primary care found that adult participants had on average a weight loss of 1.36 kg at 1-year follow-up, which is less than the 4.39 kg reduction in body mass observed at the 1-year follow up in the 2 HV group [32]. In our study, the reductions in WC in the 2 HV group may indicate a clinically significant decrease in abdominal fat. At nearly 6 cm, the decrease in WC experienced by the 2 HV intervention group at 1 year post intervention exceeded the 5 cm decrease which has been previously associated with 9 and 7% decreases in all-cause mortality in men and women, respectively [34].

Our results suggest that a home-based childhood obesity prevention intervention may improve parent weight-related outcomes while also offering beneficial effects among the entire family. Previously, we have reported the GFHS pilot intervention increased fibre and fruit intake in children, and improved body composition in children [8, 21]. This may suggest that families adjust their behaviours and their family dynamics in response to interventions which aim to prevent childhood obesity, which result in improvements in parent anthropometrics and body composition [11]. Further, it may be important to consider that these changes in parental behaviour may not be an unintended benefit of the childhood obesity intervention preventions, but rather may represent a necessary condition for preventing weight gain in children [10–12]. Thus, future programs may consider targeting the whole family in the home setting as a more efficient use of resources rather than solely targeting adults or children in a primary care or community setting. Future research should also examine the particular mechanisms by which a family-based interventions leads to changes in parents’ weight outcomes.

The majority of obesity intervention research suggests an unclear relationship between behaviour intervention dose and weight outcomes [35]. This is consistent with our results, as parents in the 2 HV, but not the 4 HV group, who had a baseline BMI ≥ 25 kg/m^2^, had significant improvements in body composition post-intervention which were maintained at one-year follow-up. While no significant reductions in weight were noted in the 4 HV group, in both weight groups for the 4 HV arm, no significant increases in weight were observed as a result of the intervention. In addition, although not statistically significant, the direction of change for most weight outcomes were in a similar direction to those among participants in the 2 HV group. This may indicate that the number of home visits is not as important as simply receiving an intervention to encourage behaviour change. Alternatively, non-significant results among the 4 HV participants may be due to lack of study power. A number of participants in the 4 HV group were excluded from the analysis due to pregnancy or breastfeeding (*n* = 10, 36% of the 28 eligible parents at the 6-month time-point), which would have reduced study power to detect a difference in this group (Fig. 1). In addition, further research is needed to determine if these results are maintained for longer periods, since previous research shows that interventions may need to be reinforced to maintain sustained results [36].

When interpreting our results, it is important to consider that this was formative, exploratory research and thus the analytic sample for each treatment group was small, increasing the possibility of type 2 errors. The sample was also primarily Caucasian drawn from Southwestern Ontario, Canada, and therefore the results may not generalize to more diverse populations. While our protocol ensured that research assistants who conducted baseline or follow-up measurements were not involved in the delivery of the intervention, lack of blinding to treatment status of participants, research assistants, and the study coordinator—who was also involved in data collection—could have led to observer bias in reporting. However, our use of objective measures for our outcomes of interest reduces the risk of reporting bias. Additionally, it may be important to consider that the design of the GFHS intervention is uniquely tailored to the needs of each family. Therefore, with this current analysis we are unable to fully elucidate which aspects of the intervention may be driving reductions in parent anthropometrics and body composition (e.g., family focus and goal setting on physical activity vs. sleep time).

## Conclusion and next steps

This study explored the effect of a home-based childhood obesity-prevention intervention on parental body composition, acknowledging the important role of addressing the whole family when implementing childhood obesity preventions. Among intervention groups, statistically and clinically significant impacts on body composition were observed among parents when stratified by weight status. These results were sustained one-year post intervention in the overweight/obese group but not in the normal weight group. This study recognizes the potential for home-based interventions to have beneficial effects not only on children, but also on the parents involved in the intervention. Future research should assess the impact of family-based childhood obesity prevention interventions on parental outcomes, considering the role of family dynamics on the success of these interventions.

## Abbreviations

%FM: Percentage fat mass
2 HV: two home visit intervention group
4 HV: four home visit intervention group
BM: Body mass
BMI: Body mass index
CI: Confidence interval
GFHS: Guelph Family Health study
WC: Waist circumference
